# Current News

**Published:** 1902-01

**Authors:** 


### Current News.

### NORTHERN ILLINOIS DENTAL SOCIETY.

THE Northern Illinois Dental Society held its fourteenth annual meeting at Joliet, October 16, 17, 1901, and elected the following officers for the ensuing year:

President, Dr. C. J. Sowle, Rockford; Vice-President, Dr.

J. E. Hancock, Joliet; Secretary, Dr. J. J. Reed, Rockford; Treasurer, Dr. M. R. Harned, Rockford; Dr. C. J. Underwood, Elgin, member of Executive Committee.

Meeting will be held at Rockford in 1902.

J. J. Reed,
*Secretary.*

---

### SOUTHERN BRANCH NATIONAL DENTAL ASSOCIATION.

The fifth annual meeting of the Southern Branch of the National Dental Association will be held at Atlanta, February 18, 1902. The Association will be in session four days. Atlanta is now the best located and equipped city in the South for holding such a meeting. This fact assures a large attendance. The South-eastern Passenger Association will give a rate of one and one-third fare for the round trip. All members are earnestly requested to be present.

C. L. Alexander,
*Corresponding Secretary S. B. N. D. A.*

Charlotte, N. C.

---

### NEW YORK ODONTOLOGICAL SOCIETY.

The thirty-fifth anniversary meeting of the New York Odontological Society will be held at the Academy of Medicine, No. 17 West Forty-third Street, New York City, January 21, 1902.

For the afternoon session, which will begin at two o'clock, an interesting list of clinics has been arranged, which will include the following:

Dr. G. W. Schwartz, of Chicago: Making and Setting Porcelain Bridge.

Dr. Geo. Evans, of New York: Demonstrating the Use of Gutta-percha Cement for setting Crowns and Bridges, and exhibiting New Instruments and Appliances for its Manipulation.

Dr. Robert Good, of Chicago: Treating a Case of Pyorrhœa and exhibiting a Case under Treatment.

Dr. Joseph Head, of Philadelphia: Demonstrating a New Method of Bleaching and Sterilizing Stained Enamel.

At this session also, Dr. Hinkins, of Chicago, will read a paper on "The Further Consideration of the Disintegration of Cements when used in or around the Teeth."

The evening meeting will take place in the large auditorium of the Academy of Medicine at eight o'clock.

The paper of the evening will be read by Dr. A. W. Harlan, of Chicago; subject, "The Basis of Dental Medicine."

W. D. TRACY,
*Corresponding Secretary.*

46 WEST THIRTY-SEVENTH STREET, NEW YORK.

---

### PENNSYLVANIA ASSOCIATION OF DENTAL SURGEONS.

THE fifty-fifth annual meeting of the Pennsylvania Association of Dental Surgeons was held October 8, 1901. The following officers were elected for the ensuing year:

President, Dr. Wilbur F. Litch; Vice-President, Dr. Eben C. Flagg; Secretary, Dr. J. Clarence Salvas; Treasurer and Librarian, Dr. Wm. H. Trueman.

The Pennsylvania Association of Dental Surgeons was organized at Philadelphia, December 16, 1845, and from that date to the present time has had a continuous existence. Dating back as it does to the early days, the dawn of organized effort for professional advancement, it is a connecting link between the past and the present. The society is one of the very few that have been sustained, and is the oldest dental society in the world. Notwithstanding the fact that the organization of other local societies drew from time to time many of its more active members, it has nevertheless continued to hold during this long period its regular stated meetings. The past year has been one of marked prosperity. There has been a large increase in membership, the meetings have been well attended, and have been made the medium for presenting papers of much practical usefulness to the profession, hence having permanent value as contributions to current dental literature.

J. CLARENCE SALVAS,
*Secretary.*

